# Death of Dr. Graves

**Published:** 1853-05

**Authors:** 


*Death of Dr. Graves.*—The death of Dr. Graves, of Dublin, will be deplored by not a few of the medical profession of this country, who are familiar with his contributions to medical knowledge. The writings of Dr. Graves have been most highly esteemed by American practitioners. From their practical character, and the internal evidence which they afford of sound judgment and sterling good sense, as well as learning and acuteness, they have exerted not a small degree of influence upon medical practice—an influence which, we believe, has generally, if not uniformly, been in the right direction.

In the following article, which we copy from the Dublin Medical Press, the editor of that able work evidently aims to present a candid notice of the professional character of the deceased. The praise bestowed is enhanced by the determination of the writer to avoid an undiscriminating and excessive strain of panegyric. The reader will not fail to observe that the superior professional ability and attainments of Dr. Graves did not secure him against injustice and neglect:

*The late Dr. Graves.*—It is not merely as a tribute of respect for the memory of Dr. Graves that we have to offer a few observations with regard to his character; the interests of our profession require that such a man should not pass from amongst us without special notice. Anxious as we are to give expression to our feelings of regret for his loss, we are doubly so in order to redeem his reputation from any stain it may have received from a fulsome eulogy furnished to a morning paper by one of the members of a fraternity which has brought discredit on our body by its unwarranted assumptions. The career of our lamented brother must not be depicted by the puffing parasite, its success must be explained by a simple statement of facts. Dr. Graves was the son of the late Dean Graves, a Fellow of Trinity College, well known to theologian, his mother having belonged to the highly respectable family of Drought, in the King's County; he therefore enjoyed the advantage of intercourse during childhood with persons of station and independence; an advantage which, in his case at least, contributed in no small degree to his subsequent position in society. He passed through college with more than the ordinary *eclat* which academic honors secure, and commenced his career as a physician some time about the year 1820: precise dates are of little importance in such a notice as this, and we therefore write from personal recollection. A residence at a German University for a sufficient time to become familiar with German habits and pursuits gave a tone to his mind which favored originality of thought and independence of action; and other causes operating in the same way at the same time, served to afford him a very fair start indeed in professional life. The Stethoscope led to the commencement of a new era in medicine, and in the hands of Dr. Wm. Stokes became a most formidable weapon in the great struggle for public patronage. Working in the same hospital the two young aspirants after medical celebrity seem to have cordially co-operated, and the result was a speedy advancement of both in reputation and practice. All kinds of extravagant nonsense have been from time to time written respecting their labors at this period; but praiseworthy as they were, we must not allow such false impressions to remain undisturbed. If we were to credit the parasite press, we should assume that, previous to this period, Dublin had never enjoyed the advantage of a physician of character. Perceval, Harvey, Cheyne, John Crampton, Barker, and a host of others, were consigned to oblivion, and no name was held up to public applause but the imitators of French and German practitioners. Even the junior members of our profession, to their subsequent cost, favored the delusion, and joined in the wild halloo against the sober conclusions of experience. It has been absurdly asserted, over and over again, by the same parasite press, that instruction by clinical lecture, was for the first time at this period introduced by Dr. Graves. Nothing could be conceived more contrary to truth; and giving the writers of such stuff ample credit for ignorance, we cannot help saying that we believe they knew and now know that the statement was a fiction. All the world knows that clinical instruction, in its most ample form, was fully employed in Dublin, Edinburgh, and London, long previous to this period. Be all this, however, as it may, out of this chaos rose the gifted object of our present notice; to our personal knowledge laughing heartily at much of what was in progress. His reputation became speedily established, and a lucrative practice followed, while his writings won for him the good opinion of the medical world. What need to enter on details. He succeeded to his heart's content, and he deserved to succeed. His loss to our profession, at the present moment, must be severely felt. A gentleman and scholar, an honest, out-spoken member of society, and a man capable of sustaining the high character he had earned, is not to be met with every day. The history of his life teaches a lesson, however; it tells us that success is not without its alloy. We believe that the subject of our remarks discovered, as others have, that the employers of medical men in this city are not what they once were. To use the political cant of the day, the "consumer" was supplanted in society by the "producer," the affluent gentleman by the hard-fisted man of business, or the ostentatious *parvenu*. A man of Dr. Graves' bearing was not suited to such a change; the low cunning and base servility, so necessary now to secure success, he could not adopt, and the consequence we believe was, that his practice for some years had not been so extensive as formerly. He had, however, "made hay while the sun shone," and any falling off in this way gave him no trouble. Another page in his history is also instructive, and let the neglected man of ability study it. Wealth, connection, and high character, will sometimes no more secure its reward than the humble, patient, pursuit of knowledge. If ever man had fairly won a Professorship, Dr. Graves had won that called the Regius Professorship of Physic in Trinity College, yet it was denied him. We cannot, perhaps, correctly say that "an eagle towering in his pride of place was by a mousing owl hawked at and killed," but it was something like it. Premiums, certificates, gold medals, and all other academic honors, were forgotten, as if to show the estimation in which they were held by those who dispensed them; and the first medical prize in the University was awarded to one who, in this respect at least, had no claim whatever to any such distinction. Our limits do not permit us to offer to our readers more than this very inadequate sketch of the life of this distinguished man, but the majority of them can supply from their own sources of information much of what we have been obliged to pass over.

